# Mapping Australia’s hospital care for malignant neoplasms of the lip, oral cavity, and pharynx: An ecological study

**DOI:** 10.1097/MD.0000000000047149

**Published:** 2026-01-09

**Authors:** Mohammed I. Alsaif, Abdullah S. Bin Rahmah, Abdallah Y. Naser

**Affiliations:** a Department of Periodontics and Community Dentistry, College of Dentistry, King Saud University, Riyadh, Saudi Arabia; b Department of Applied Pharmaceutical Sciences and Clinical Pharmacy, Faculty of Pharmacy, Isra University, Amman, Jordan.

**Keywords:** Australia, cancer, hospitalization, malignancy, oral cavity

## Abstract

Neoplastic malignant tumors originating from inside the upper aerodigestive tract are referred to as lip, oral cavity, and pharyngeal malignancies. This study aimed to analyze the hospitalization profile for malignant neoplasms of the lip, oral cavity, and pharynx in Australia. This was a longitudinal ecological study that utilized the National Hospitals Data Collection in Australia database between 1998 and 2023. Hospitalization data were identified using the ICD codes C00-C14. Between 1998 and 2023, Australia reported a total of 160,055 hospitalization episodes. A large proportion of these admissions were overnight stays (69.7%). Most hospitalizations occurred among males (71.9%). The highest proportion of hospitalization episodes was amongst the 60 to 74 age group, who accounted for 39.8% of total episodes. Malignant neoplasm of other and unspecified parts of the tongue was the most common hospitalization reason, accounting for 14.5% of episodes. During the study period, the rate of same-day hospitalization increased by 55.9% (*P* < .001). However, the rates of overnight-stay-related hospitalization decreased by 9.7% (*P* < .001). Malignant neoplasms of the lip, oral cavity and pharynx are responsible for a sizeable healthcare burden among Australians, with the highest proportion of hospitalized cases occurring for males and those aged 60 to 74 years. There has been a significant increase in hospitalization involving same-day procedures. Investment in models of care offering same-day treatment and supportive care for older adults may contribute to mitigating inpatient pressures.

## 1. Introduction

A broad range of neoplastic malignant tumors originating from places inside the upper aerodigestive tract are referred to as lip and oral cavity, and pharyngeal malignancies.^[1]^ Globally significant and deadly, oral cancer, mostly squamous cell carcinoma (SCC) originating from the mouth lining, is becoming more common. SCC develops on the lip, in the tonsils, oropharynx, oral cavity, nasopharyngeal, laryngeal, and salivary gland malignancies.^[2]^

Including malignancies of the tongue, lips, and mouth, lip and oral cavity cancers rank as the 16th most prevalent neoplasm globally, accounting for around 177,000 fatalities and nearly 355,000 new diagnoses in 2018.^[3]^ Several risk factors are strongly associated with lip and oral cavity and pharynx cancer, such as tobacco, which is reported in a large percentage of these cancer cases. Other risk factors include alcohol consumption and human papillomavirus (HPV) infection.^[3]^

Recent studies from Australia reported a reduction in lip cancer incidence, while other cancers, such as tonsil and oropharyngeal SCCs, significantly increased.^[4]^ The incidence rate of lip-oral cavity and pharynx cancers increased 1.3 times from 1999 to 2008 and 2000 to 2018 among Aboriginal and Torres Strait Islander peoples living in Australia.^[1]^ Another study analyzing data from several countries reported that the highest incidence rate was seen in Australia (4.3 in males, 1.2 in females).^[2]^

Although the mortality rates due to cancer in Australia remain within a close range (3 out of 10), new case diagnosis and hospitalization rates vary across states. Regarding oral SCC, specific information on clinicopathological presentation and clinical outcome data is still limited in the literature.^[4]^ Compared to other developed countries, for instance, the mortality rates in Canada between 1992 and 2010 were 13.35 deaths per million individuals per year for oral SCC cancer.^[5]^ In Central and Eastern Europe, the projected age-standardized cancer death rate for men was 5.1 in 2012, while in Northern Europe, it was 1.6.^[6]^ Additionally, hospitalization rates vary among countries. In Brazil, between 2007 and 2016, the number of hospitalizations for oral cavity cancer and oropharyngeal cancer were 52,799 and 34,516, respectively.^[7]^ This study aimed to examine the hospitalization profile for malignant neoplasms of the lip-oral cavity and pharynx in Australia.

## 2. Material and methods

### 2.1. Study design and population

This was a longitudinal ecological study that utilized publicly available hospitalization data in Australia between 1998 and 2023. Patients hospitalized due to malignant neoplasms of the lip-oral cavity and pharynx in all private and public hospitals are recorded.^[8]^

### 2.2. Data sources

#### 2.2.1. The National Hospital Morbidity database

Data for this study was collected from the National Hospitals Data Collection in Australia.^[9]^ This database was previously utilized to examine the epidemiology of various health conditions in Australia.^[10–12]^ The data included information about the patients’ age, gender, hospitalization cause, and hospitalization type. The National Hospitals Data Collection includes hospitalization episodes for patients admitted to hospitals from all institutions in Australia. Emergency patients may be admitted straight to a specialized area or through the emergency department (ED) (i.e., a critically ill patient who arrives at a hospital ED via an ambulance and is taken directly to the intensive care unit). If a patient has a condition that does not require evaluation and treatment within 24 hours, they are admitted on an elective basis.^[13]^

#### 2.2.2. Australian Bureau of Statistics

Mid-year population data from the Australian Bureau of Statistics was used to estimate the hospitalization rate.^[14–16]^

### 2.3. Statistical analysis

All analyses were performed using SPSS version 29 (IBM Corp, Armonk). Hospitalization data were identified using the following ICD codes C00-C14. Hospitalization rates were estimated at 95% confidence intervals (CIs). We assessed the variation in hospitalization rates using the Pearson chi-square test for independence.

## 3. Results

### 3.1. Total hospitalization numbers

Between 1998 and 2023, Australia reported a total of 160,055 hospitalization episodes. Over this period, the annual number of these hospitalizations increased by 51.8%, from 5397 to 8194 (*P* < .001). A large proportion of these admissions were overnight stays (69.7%). Most hospitalizations occurred among males (71.9%). The highest proportion of hospitalization was seen in the 60 to 74 and 15 to 59 age groups, respectively accounting for 39.8% and 39.7% of the total number of hospitalizations (Table 1).

**Table 1:** Total hospitalization episodes for malignant neoplasms of lip, oral cavity, and pharynx from 1998 to 2023 in Australia.

Category	Number of episodes	% from total
Hospitalization by type of admissions		
Overnight-stay	111,522	69.7
Same day	48,533	30.3
Hospitalization by gender		
Males	115,087	71.9
Females	44,966	28.1
Hospitalization by age groups		
Below 15 yr	1326	0.8
15–59 yr	63,584	39.7
60–74 yr	63,736	39.8
75 yr and above	31,408	19.6

### 3.2. Hospitalizations profile

Malignant neoplasm of other and unspecified parts of tongue was the most common hospitalization reason, accounting for 14.5% of total related admissions, followed by malignant neoplasms of “tonsil” with 13.3%, “base of tongue” with 12.7%, and “lip” with 10.1% (Table 2).

**Table 2:** Percentage of hospitalization from total number of admissions per ICD code.

ICD code	Description	% from total number of admissions
C00	Malignant neoplasm of lip	10.1
C01	Malignant neoplasm of base of tongue	12.7
C02	Malignant neoplasm of other and unspecified parts of tongue	14.5
C03	Malignant neoplasm of gum	2.6
C04	Malignant neoplasm of floor of mouth	6.0
C05	Malignant neoplasm of palate	3.5
C06	Malignant neoplasm of other and unspecified parts of mouth	5.3
C07	Malignant neoplasm of parotid gland	8.3
C08	Malignant neoplasm of other and unspecified major salivary glands	1.7
C09	Malignant neoplasm of tonsil	13.3
C10	Malignant neoplasm of oropharynx	4.7
C11	Malignant neoplasm of nasopharynx	6.7
C12	Malignant neoplasm of piriform sinus	4.1
C13	Malignant neoplasm of hypopharynx	3.2
C14	Malignant neoplasm of other and ill-defined sites in the lip, oral cavity and pharynx	3.3

### 3.3. Total hospitalization rates

**Figure 1.**
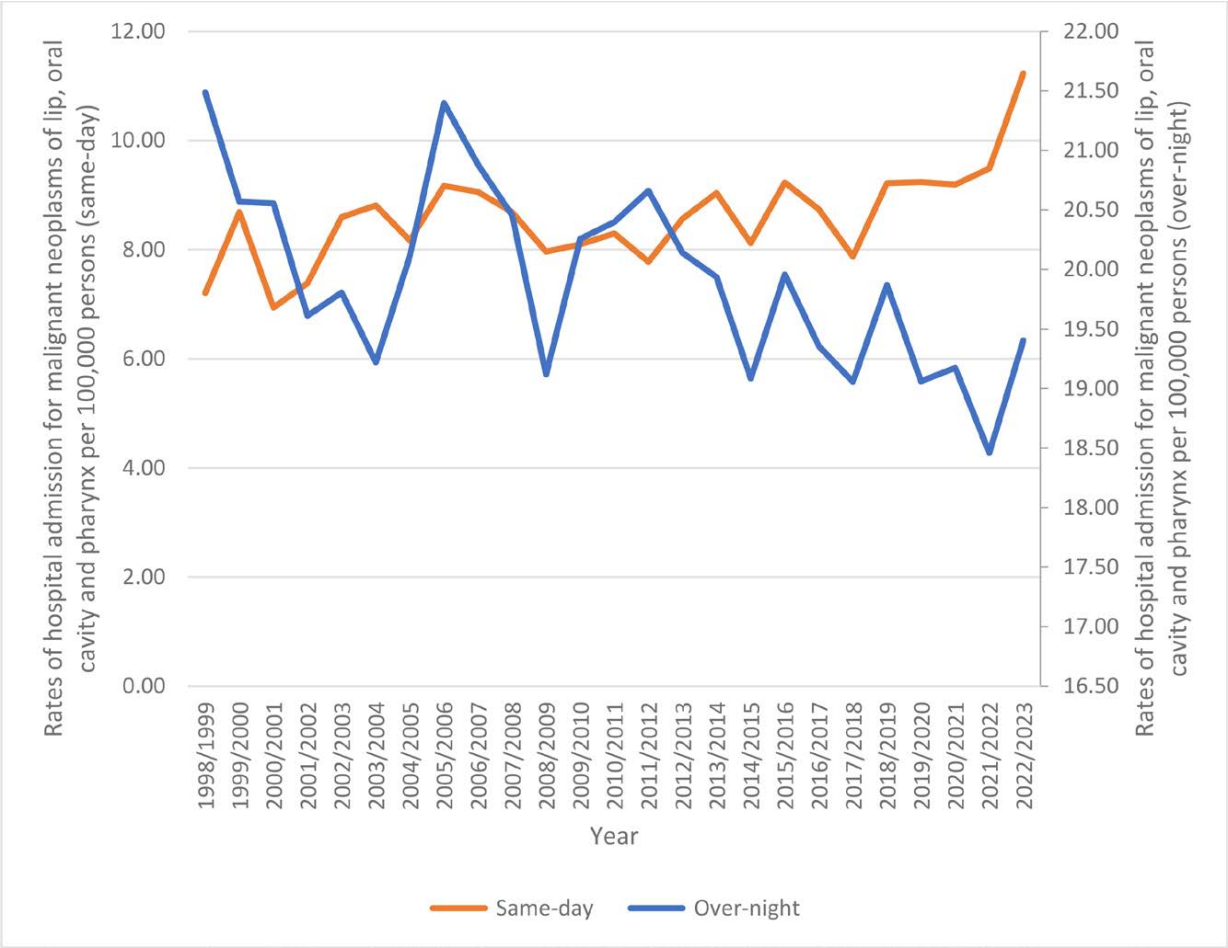
Total rates of same-day and overnight-stay patients’ hospitalization for malignant neoplasms of lip, oral cavity and pharynx in Australia between 1998 and 2023.

**Figure 2.**
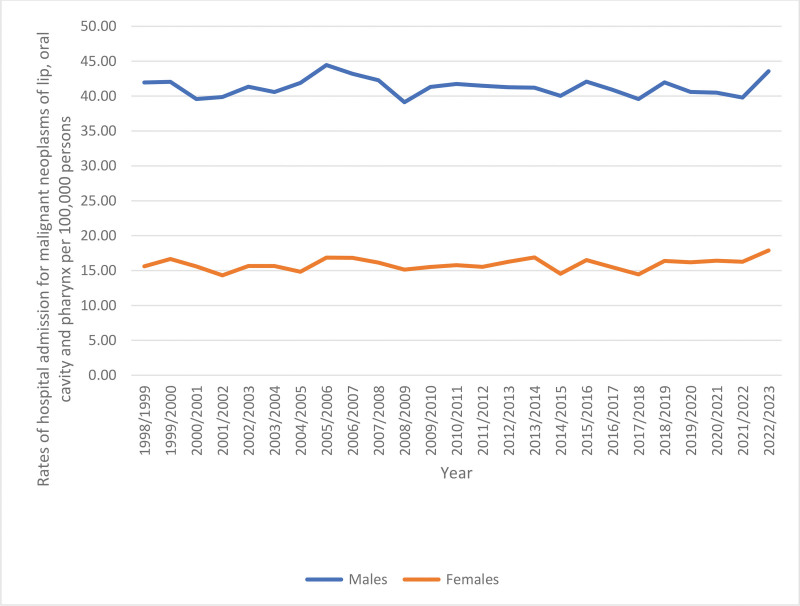
Total hospitalization rates for malignant neoplasms of lip, oral cavity and pharynx in Australia stratified by gender.

**Figure 3.**
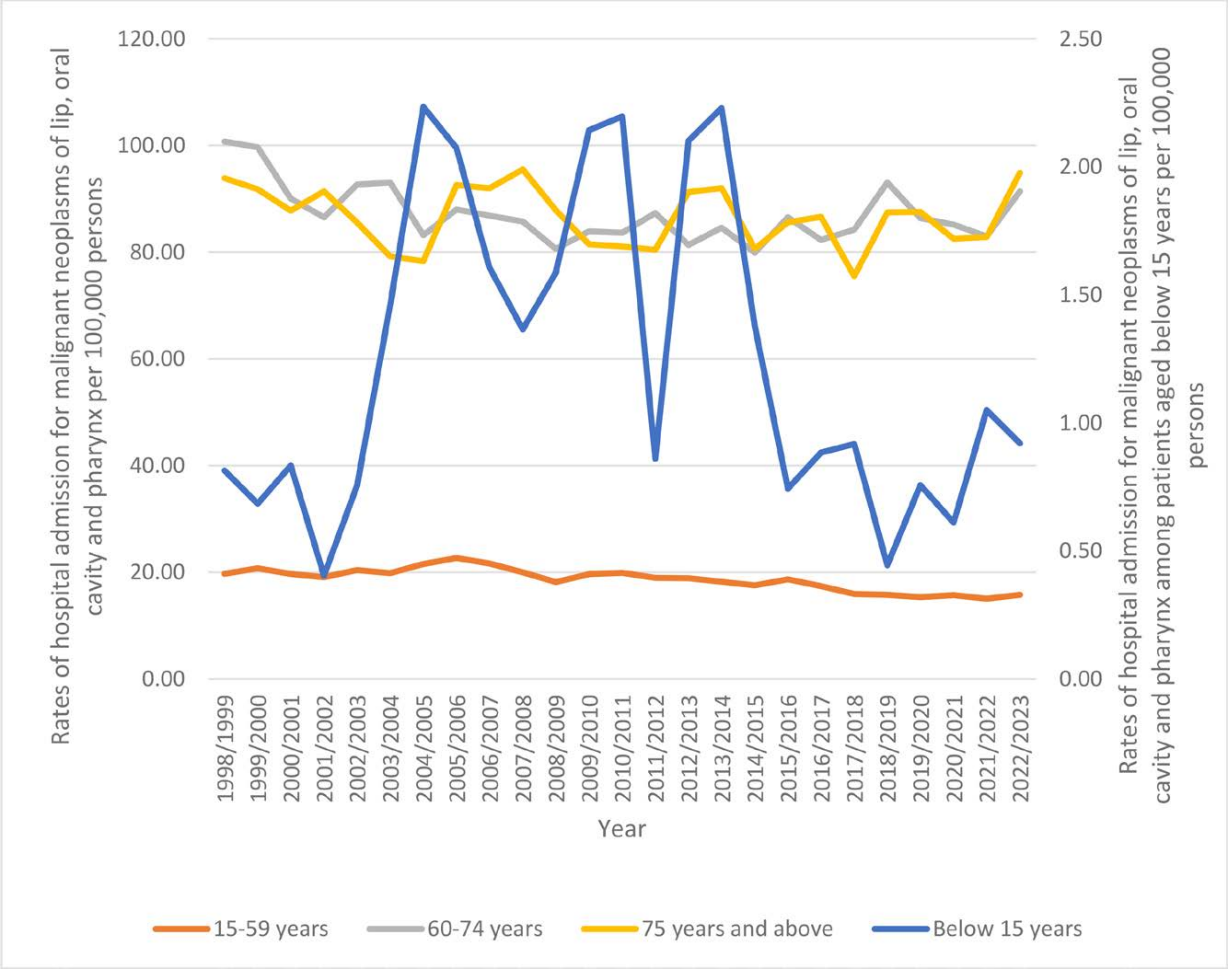
Total hospitalization rates for malignant neoplasms of lip, oral cavity and pharynx in Australia stratified by age group.

Throughout the study, the total hospitalization rate increased by 7.2% from 28.69 (95% CI 27.92–29.45) per 100,000 persons in 1998 to 30.74 (95% CI 30.08–31.41) in 2023, (*P* < .001). The rates of same-day hospitalization increased by 55.9% (*P* < .001). However, the rates of overnight-stay-related hospitalization decreased by 9.7% (*P* < .001) (Table 3, Fig. 1). The hospitalization rate increased by 3.8% among males, (*P* < .01) and by 14.6% among females (*P* < .001) (Table 3, Fig. 2). Moreover, hospitalization rates showed a decline across the 60 to 74 and 15 to 59 age groups by 19.9% (*P* < .001) and 9.3% (*P* < .001), respectively. In comparison, the rate increased among patients below 15 years and those aged 75 years and above by 13.0% and 1.1%, respectively, (*P* > .05) (Table 3, Fig. 3).

**Table 3:** Percentage change in the total hospitalization rates stratified by category.

Category	Hospitalization rate in 1998 per 100,000 persons (95% CI)	Hospitalization rate in 2023 per 100,000 persons (95% CI)	% change from 1998 to 2023
Hospitalization rates by type of admissions			
Overnight-stay	21.49 (20.82–22.15)	19.40 (18.88–19.93)	−9.7
Same day	7.20 (6.82–7.59)	11.23 (10.83–11.63)	55.9
Hospitalization rates by gender			
Males	41.96 (40.65–43.27)	43.56 (42.44–44.69)	3.8
Females	15.60 (14.81–16.40)	17.88 (17.17–18.60)	14.6
Hospitalization rates by age groups			
Below 15 yr	0.81 (0.53–1.10)	0.92 (0.65–1.19)	13.0
15–59 yr	19.72 (18.92–20.52)	15.79 (15.17–16.41)	−19.9
60–74 yr	100.75 (96.42–105.07)	91.39 (88.43–94.35)	−9.3
75 yr and above	93.87 (87.93–99.81)	94.88 (90.69–99.07)	1.1

CI = confidence interval.

### 3.4. Hospitalization rates profile

**Figure 4.**
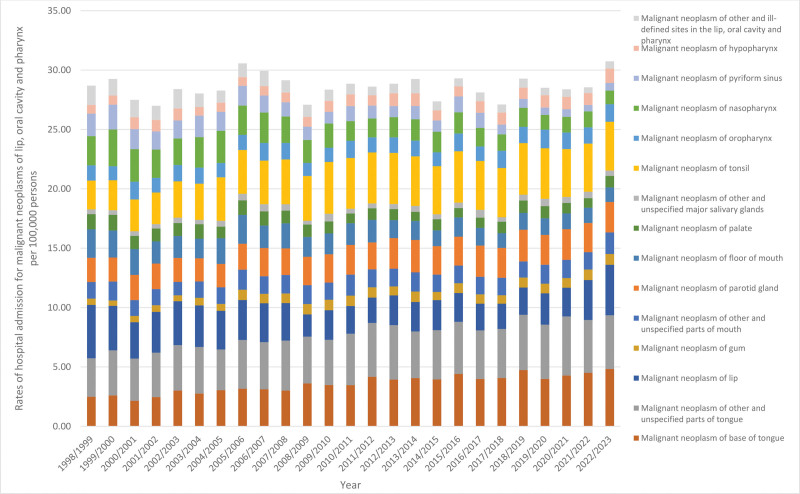
Rates of hospitalization for malignant neoplasms of lip, oral cavity and pharynx in Australia between 1998 and 2023.

During the study period, “base of tongue,” “gum,” “tonsil,” “hypopharynx,” “other and unspecified parts of tongue,” “other and unspecified parts of mouth,” “parotid gland,” “oropharynx,” and “other and unspecified major salivary glands” all saw increases as reasons for hospitalization by 94.2%, 71.8%, 69.5%, 63.5%, 39.4%, 30.2%, 25.5%, 15.1% (*P* < .001), and 12.4% (*P* > .05), respectively. Conversely, “pyriform sinus,” “other and ill-defined sites in the lip, oral cavity and pharynx,” “nasopharynx,” “floor of mouth,” “palate,” and “lip” saw decrease as reasons for hospitalization by 66.0%, 62.3%, 53.1%, 48.8%, 23.8%, and 5.5%, respectively, (*P* < .001) (Table 4, Fig. 4).

**Table 4:** Percentage change in the hospitalization rates for malignant neoplasms of lip, oral cavity, and pharynx from 1998 to 2023 in Australia.

Malignant neoplasms	Hospitalization rate in 1998 per 100,000 persons (95% CI)	Hospitalization rate in 2023 per 100,000 persons (95% CI)	% change from 1998 to 2023
Malignant neoplasm of lip	4.50 (4.19–4.80)	4.25 (4.00–4.50)	−5.5
Malignant neoplasm of base of tongue	2.49 (2.26–2.71)	4.83 (4.57–5.10)	94.2
Malignant neoplasm of other and unspecified parts of tongue	3.24 (2.99–3.50)	4.52 (4.27–4.78)	39.4
Malignant neoplasm of gum	0.53 (0.42–0.63)	0.90 (0.79–1.02)	71.8
Malignant neoplasm of floor of mouth	2.40 (2.18–2.62)	1.23 (1.09–1.36)	−48.8
Malignant neoplasm of palate	1.28 (1.11–1.44)	0.97 (0.85–1.09)	−23.8
Malignant neoplasm of other and unspecified parts of mouth	1.40 (1.23–1.57)	1.82 (1.66–1.98)	30.2
Malignant neoplasm of parotid gland	2.04 (1.84–2.25)	2.56 (2.37–2.75)	25.5
Malignant neoplasm of other and unspecified major salivary glands	0.40 (0.31–0.49)	0.45 (0.37–0.53)	12.4
Malignant neoplasm of tonsil	2.42 (2.20–2.65)	4.11 (3.86–4.35)	69.5
Malignant neoplasm of oropharynx	1.28 (1.12–1.44)	1.47 (1.33–1.62)	15.1
Malignant neoplasm of nasopharynx	2.47 (2.24–2.69)	1.16 (1.03–1.28)	−53.1
Malignant neoplasm of piriform sinus	1.89 (1.69–2.08)	0.64 (0.55–0.74)	−66.0
Malignant neoplasm of hypopharynx	0.74 (0.62–0.86)	1.21 (1.08–1.34)	63.5
Malignant neoplasm of other and ill-defined sites in the lip, oral cavity, and pharynx	1.62 (1.44–1.80)	0.61 (0.52–0.71)	−62.3

CI = confidence interval.

### 3.5. Hospitalization rates by gender

**Figure 5.**
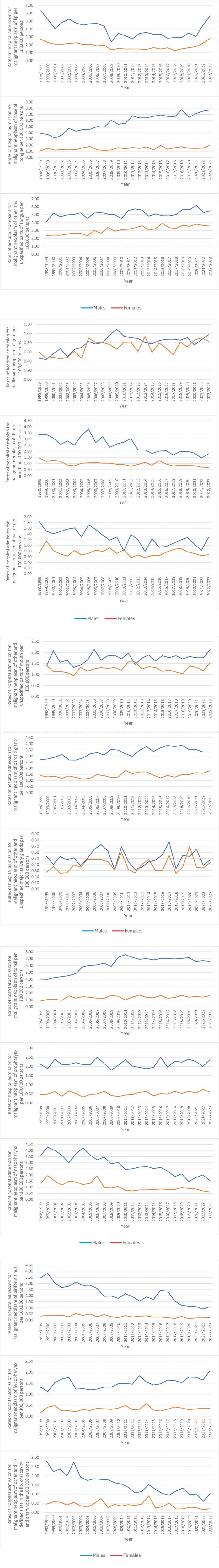
Rates of hospitalization by gender.

Hospitalization rates were higher among males than females (Fig. 5).

### 3.6. Hospitalization rates by age

**Figure 6.**
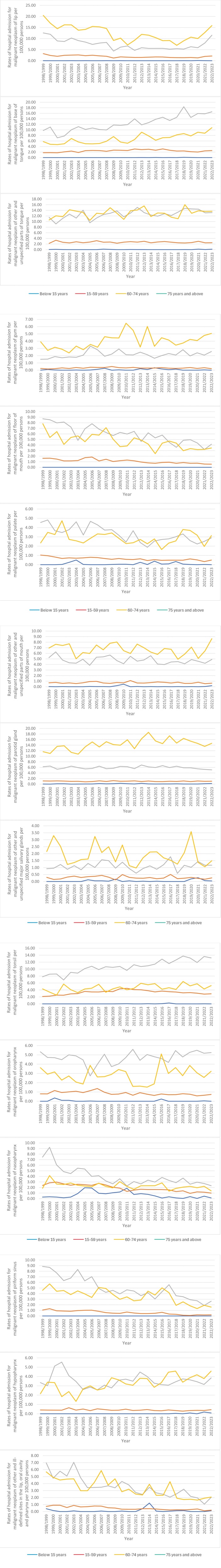
Rates of hospitalization by age group.

Hospitalization rates were higher in patients aged 60 years and older (Fig. 6).

## 4. Discussion

The overall hospitalization rate increased by 7.2%, from 28.69 per 100,000 persons in 1998 (95% CI 27.92–29.45) to 30.74 in 2023 (95% CI 30.08–31.41) (*P* < .001). This trend contrasts with research conducted in Brazilian hospitals between January 2018 and August 2021, which showed a reduction in the hospitalization rate compared to previous years by around 18.4%. This reduction in the hospitalization rate among cancer patients may be attributed to the effects of COVID-19 pandemic, which affected hospitalization rate for various diseases, despite an increase in the incidence of cancers during the same period.^[17]^ Additionally, a study from Spain conducted between 2009 and 2019 showed a decrease in the hospitalization rate for head and neck cancers, including lip and oral cavity cancer.^[18]^ The study from Spain showed a decrease in hospitalization rate for all diagnoses except for tonsil cancer. Although it also showed a 1.4-fold increase in oral cavity and laryngeal cancer amongst females.^[18]^ The difference between our findings and those from Spain may be due to variations in sample characteristics. Multiple factors might have contributed to the observed differences in findings between our research and other international studies. These could include differences in study design, the study time frame, ICD codes used, and variations in healthcare policies across countries.

### 4.1. Gender differences

A large proportion of hospital admissions were overnight stays (69.7%), with most hospitalizations occurring among males (71.9%). The Global Epidemiological Study supports these findings, and showed that approximately 70% of newly hospitalized cases are for males.^[3]^ This is likely due to risk factors such as tobacco and alcohol use, which are more prevalent among males. These risk factors contribute to the observed increase in the hospitalization rates in males. Socioeconomic factors, among males such as the use of cigarettes and alcohol, play an important role in the increased incidence of oral cancer. Tobacco use is a well-known risk factor, significantly increasing the risk of developing oral cavity cancer, which is more prevalent in males compared to females.^[19,20]^

In addition to the important role of gender on the observed hospitalization pattern, age is also one of the most important factors shaping hospitalization patterns for a wide-range of medical conditions, including oral cavity-related neoplasms.

### 4.2. Age distribution

In terms of age distribution, the highest proportion of hospitalization was seen in the 60 to 74 and 15 to 59 age groups, accounting for 39.8% and 39.7% of the total number of hospitalizations, respectively. These findings align with research conducted among Aboriginal and Torres Strait Islander people in Australia, which also demonstrated a higher incidence of lip and oral cavity, and pharynx cancer amongst these groups compared to young people.^[1]^ Similar to a study conducted in Canada, their findings showed a positive relationship between age and incidence of oral cancers, with an incidence peak at age 50 to 59 years.^[5]^ This consistency across studies may be explained by age-related comorbidities and challenges in treating elderly patients. Elderly patients are at high risk of comorbidities, which increase the likelihood of hospitalization and adverse reactions to chemotherapy. These complications can lead to early discontinuation of cancer treatment, as the side effects may become intolerable.^[21–23]^

### 4.3. Hospitalization patterns

While no previous studies were found directly comparing trends around overnight-stay hospitalizations, our results indicate a significant shift in hospitalization patterns. The rates of same-day hospitalization increased by 55.9%. However, the rates of overnight-stay-related hospitalization decreased by 9.7%. This decrease in overnight stays could be due to improvements in recovery times and the health care system. Some patients may require tumor resection procedures, such as pharyngolaryngectomy, which are associated with higher postoperative complications, especially in elderly patients with comorbidities. Conditions such as malnutrition and respiratory issues often require overnight stays for close monitoring and recovery support.^[17]^

### 4.4. Preventive strategies

In this research, hospitalization rates were higher among males than among females. The hospitalization rate increased by 3.8% among males and by 14.6% among females. An epidemiological study reported increasing trends in oral and pharyngeal cancer for both males and females in Australia.^[24]^ This may be affected by the risk factors such as smoking tobacco, which decreased over the time period among males but increased among females.^[25]^

The most common reason for hospitalization was malignant neoplasm of other and unspecified parts of the tongue, accounting for 14.5% of the total related admissions. This was followed by malignant neoplasms of the tonsil (13.3%), base of tongue (12.7%), and lip (10.1%). In contrast, a separate study showed that lip and oral cavity cancers represented 57.4% of the total 678,900 incident cases.^[26]^ This difference may be due to the differences in sample characteristics between the 2 studies. This highlights the importance of implementing preventive medical and behavioral interventions. To effectively lower the incidence rate of lip and oral cavity, and pharynx cancers, it is essential to focus on modifiable risk factors such as tobacco and alcohol use. Enhancing comprehensive programs that educate the public about these risks may reduce overall incidence and hospitalization rates, which will lead to a decrease in treatment costs. Additionally, enhancing vaccination programs to include the HPV virus would further decrease incidence rates. Future research should prioritize these strategies and assess their impact on hospitalization rates to improve patient outcomes.

### 4.5. Study limitations

This study has several limitations. First, our analysis focused on Australia; including data from other countries will improve the generalizability of our findings. Second, while we focused on the hospitalization rate, including associated risk factors such as HPV or tobacco use, could offer a more comprehensive understanding of the results. Ecological studies analyze population-level data, not individual-level outcomes, which means that causality cannot be inferred and observed trends may not be explained. Ecological fallacy risk is common in this study design, by assuming that group-level trends apply to individuals. Moreover, we were not able to adjust for important confounding variables such as smoking, alcohol use, HPV status, socioeconomic status, or geographic variation. Therefore, the study findings should be interpreted carefully. Finally, most studies discussed in the literature study the incidence rate rather than hospitalization patterns, which may impact our results.

## 5. Conclusion

Malignant neoplasms of the lip and oral cavity and pharynx are responsible for a sizeable healthcare burden among Australians, with the highest proportion of hospitalized cases occurring for males and those aged 60 to 74 years. There has been a significant increase over time in hospitalizations involving same-day procedures. The most common principal diagnoses for these episodes are neoplasms of the tongue. Such increases will be offset only by expanded screening programs for early detection, coupled with focused prevention efforts targeting high-risk populations, especially older males. Ongoing research into the causes of tongue cancers, coupled with monitoring trends in hospitalizations, will better prepare the healthcare system to manage resources in the future. Investment in models of care offering same-day treatment and supportive care for older adults may mitigate inpatient pressures.

## Author contributions

**Conceptualization:** Mohammed I. Alsaif, Abdullah S. Bin Rahmah.

**Data curation:** Mohammed I. Alsaif, Abdallah Y. Naser.

**Formal analysis:** Mohammed I. Alsaif, Abdallah Y. Naser.

**Funding acquisition:** Mohammed I. Alsaif.

**Investigation:** Mohammed I. Alsaif, Abdullah S. Bin Rahmah, Abdallah Y. Naser.

**Methodology:** Mohammed I. Alsaif.

**Project administration:** Mohammed I. Alsaif.

**Resources:** Mohammed I. Alsaif, Abdullah S. Bin Rahmah, Abdallah Y. Naser.

**Software:** Mohammed I. Alsaif, Abdallah Y. Naser.

**Supervision:** Mohammed I. Alsaif.

**Validation:** Mohammed I. Alsaif, Abdullah S. Bin Rahmah.

**Visualization:** Mohammed I. Alsaif, Abdullah S. Bin Rahmah.

**Writing – original draft:** Mohammed I. Alsaif, Abdullah S. Bin Rahmah, Abdallah Y. Naser.

**Writing – review & editing:** Mohammed I. Alsaif, Abdullah S. Bin Rahmah, Abdallah Y. Naser.

## References

[1] Sethi S Ju X Logan R Hedges J Garvey G Jamieson L. Lip, oral and oropharyngeal cancer incidence among aboriginal and torres strait islander peoples: first report from Australian population-based cancer registry, 1999-2018. Aust Dent J. 2024;69:182–8. 38469883 10.1111/adj.13013

[2] Miranda-Filho A Bray F. Global patterns and trends in cancers of the lip, tongue and mouth. Oral Oncol. 2020;102:104551. 31986342 10.1016/j.oraloncology.2019.104551

[3] Hernández-Morales A González-López BS Scougall-Vilchis RJ. Lip and oral cavity cancer incidence and mortality rates associated with smoking and chewing tobacco use and the human development index in 172 countries worldwide: an ecological study 2019-2020. Healthcare (Basel). 2023;11:1063. 37107897 10.3390/healthcare11081063 PMC10137392

[4] Sun A Sharma D Choi S-W Ramamurthy P Thomson P. Oral cancer in Australia: Rising incidence and worsening mortality. J Oral Pathol Med. 2023;52:328–34. 36852511 10.1111/jop.13421

[5] Ghazawi FM Lu J Savin E. Epidemiology and patient distribution of oral cavity and oropharyngeal SCC in Canada. J Cutan Med Surg. 2020;24:340–9. 32238063 10.1177/1203475420915448

[6] Diz P Meleti M Diniz-Freitas M. Oral and pharyngeal cancer in Europe: Incidence, mortality and trends as presented to the global oral cancer forum. Transl Res Oral Oncol. 2017;2:2057178X17701517.

[7] Faria SO Nascimento MCD Kulcsar MAV. Malignant neoplasms of the oral cavity and oropharynx treated in Brazil: what do hospital cancer records reveal? Braz J Otorhinolaryngol. 2022;88:168–73. 32682819 10.1016/j.bjorl.2020.05.019 PMC9422503

[8] Australian Institute for Health and Welfare. Principal diagnosis data cubes. 2022. https://www.aihw.gov.au/reports/hospitals/principal-diagnosis-data-cubes/contents/data-cubes. Accessed February 14, 2025.

[9] Australian Institute for Health and Welfare. National Hospitals Data Collection. 2019. https://www.aihw.gov.au/about-our-data/our-data-collections/national-hospitals. Accessed February 14, 2025.

[10] Alrajeh A Naser AY Aldabayan YS. Hospitalisation patterns for respiratory diseases in Australia: an ecological study. BMJ Open. 2024;14:e084286. 10.1136/bmjopen-2024-084286 PMC11603736 39608993

[11] Hassanin FF Naser AY Aalam WA Hanbazazh M. Eye and adnexa hospitalization in Australia: an ecological study. Medicine (Baltim). 2024;103:e38829. 10.1097/MD.0000000000038829 PMC11224824 38968452

[12] Naqeeb MR Naser AY. Hospitalisation trends for choroid and retina diseases in the Past 20 Years: an ecological study in Australia. Clin Optom (Auckl). 2023;15:247–59. 37868141 10.2147/OPTO.S433266 PMC10590135

[13] Department of Health. Annual Report 2021–22. 2022. https://www.healthywa.wa.gov.au/~/media/Corp/Documents/Reports-and-publications/Annual-report/2022/DOH-annual-report.pdf. Accessed February 14, 2025.

[14] Australian Bureau of Statistics. About. 2023. https://www.abs.gov.au/about. Accessed February 14, 2025.

[15] Australian Bureau of Statistics. Historical population. 2019. https://www.abs.gov.au/statistics/people/population/historical-population/latest-release. Accessed February 14, 2025.

[16] Australian Bureau of Statistics. National, state and territory population. 2022. https://www.abs.gov.au/statistics/people/population/national-state-and-territory-population/latest-release. Accessed February 14, 2025.

[17] Cunha ARD Velasco SRM Hugo FN Antunes JLF. Hospitalizations for oral and oropharyngeal cancer in Brazil by the SUS: impacts of the covid-19 pandemic. Rev Saude Publica. 2023;57(suppl 1):3s. 37255114 10.11606/s1518-8787.2023057004708 PMC10185317

[18] Carazo-Casas C Gil-Prieto R Hernández-Barrera V Gil de Miguel A. Trends in hospitalization and death rates among patients with head and neck cancer in Spain, 2009 to 2019. Hum Vaccin Immunother. 2022;18:2082192. 35930449 10.1080/21645515.2022.2082192 PMC9621082

[19] Gharat SA Momin M Bhavsar C. Oral squamous cell carcinoma: current treatment strategies and nanotechnology-based approaches for prevention and therapy. Crit Rev Ther Drug Carrier Syst. 2016;33:363–400. 27910740 10.1615/CritRevTherDrugCarrierSyst.2016016272

[20] Sundermann BV Uhlmann L Hoffmann J Freier K Thiele OC. The localization and risk factors of squamous cell carcinoma in the oral cavity: a retrospective study of 1501 cases. J Craniomaxillofac Surg. 2018;46:177–82. 29242026 10.1016/j.jcms.2017.10.019

[21] Grønberg BH Sundstrøm S Kaasa S. Influence of comorbidity on survival, toxicity and health-related quality of life in patients with advanced non-small-cell lung cancer receiving platinum-doublet chemotherapy. Eur J Cancer. 2010;46:2225–34. 20471248 10.1016/j.ejca.2010.04.009

[22] Neugut AI Matasar M Wang X. Duration of adjuvant chemotherapy for colon cancer and survival among the elderly. J Clin Oncol. 2006;24:2368–75. 16618946 10.1200/JCO.2005.04.5005

[23] Wenkstetten-Holub A Fangmeyer-Binder M Peter F. Prevalence of comorbidities in elderly cancer patients. memo. Magazine Eur Med Oncol. 2020;14:15.

[24] Derbi H Kruger E Tennant M. Incidence of oral cancer in Western Australia (1982-2009): Trends and regional variations. Asia Pacific J Clin Oncol. 2014;12:305. 10.1111/ajco.12205 24935669

[25] OECD Library. Health at a Glance 2011. 2011. https://www.oecd-ilibrary.org/social-issues-migration-health/health-at-a-glance-2011_health_glance-2011-en. Accessed February 14, 2025.

[26] Du M Nair R Jamieson L Liu Z Bi P. Incidence trends of lip, oral cavity, and pharyngeal cancers: global burden of disease 1990-2017. J Dent Res. 2020;99:143–51. 31874128 10.1177/0022034519894963

